# Mapping and characterization of quantitative trait loci for mesocotyl elongation in rice (*Oryza sativa* L.)

**DOI:** 10.1186/1939-8433-5-13

**Published:** 2012-06-26

**Authors:** Hyun-Sook Lee, Kazuhiro Sasaki, Atsushi Higashitani, Sang-Nag Ahn, Tadashi Sato

**Affiliations:** 1 Graduate School of Life Sciences, Tohoku University, 2-1-1 Katahira, Aoba-ku, Sendai, 980-8577 Japan; 2 College of Agriculture and Life Sciences, Chungnam National University, Daejeon, 305-764 South Korea; 3 Plant Breeding, Genetics and Biotechnology Division, International Rice Research Institute, DAPO, Box 7777, Metro Manila, Philippines

**Keywords:** Rice (*Oryza sativa* L.), Chromosome segment substitution line (CSSL), Direct-seeding, Mesocotyl elongation, Quantitative trait locus (QTL)

## Abstract

Mesocotyl elongation is an important trait for seedling emergence in direct-seeding cultivation in rice. In this study, a backcross inbred line (BIL) population from a cross between Kasalath and Nipponbare was employed to map quantitative trait loci (QTLs) for mesocotyl elongation. A total of 5 QTLs for mesocotyl length were identified on chromosomes 1, 3, 7, 9, and 12 in 2 independent experiments. At all QTL, the Kasalath alleles contributed to an increase in mesocotyl length. Two QTLs (*qMel-1* and *qMel-3*) on chromosomes 1 and 3 were consistently detected in both experiments. To fine map the QTLs, a cross was made between 2 chromosome segment substitution lines (CSSL-6 and CSSL-15), each harboring the Kasalath allele across the *qMel-1* and *qMel-3* regions, and an F_2:3_ population was developed. A two-way ANOVA indicated that no epistatic interaction was detected between the 2 QTLs in the F_2_ population (*P* = 0.31). Moreover, analysis of two F_3_ near-isogenic lines (NILs) derived from the same cross, indicated that the 2 QTLs act additively in distinct or complementary pathways in controlling mesocotyl elongation. Substitution mapping indicated that the *qMel-1* QTL was located between the 2 SSR markers RM5448 and RM5310, which are 3,799-kb apart, and that the *qMel-3* QTL was located between the 2 SSR markers RM3513 and RM1238, which are 6,964-kb apart. To our knowledge, this is the first report to fine-map QTLs for mesocotyl elongation and to analyze their interaction.

**Electronic supplementary material:**

The online version of this article (doi:10.1186/1939-8433-5-13) contains supplementary material, which is available to authorized users.

## Background

In rice, direct-seeding cultivation is becoming popular in Korea and Japan, because it requires less labor relative to transplanting one. The mesocotyl is an embryonic structure between the scutellar node and coleoptilar node and is directly related to rice seedling emergence, since it elongates during germination to push the shoot tip above the soil surface. However, poor emergence and inadequate stand establishment of seedlings caused by short mesocotyls can lead to yield loss in direct seeding cultivation.

Mesocotyl elongation displays a large variation among rice germplasm. The mesocotyl of *indica* cultivars is longer than that of *japonica* cultivars (Takahashi [1978]), and the variation in mesocotyl length in *indica* cultivars is larger than that of *japonica* cultivars (Hamada [1937]; Takahashi [1978]). Upland rice cultivars display a longer mesocotyl and a higher proportion of elongated mesocotyls compared to the lowland cultivars (Chang and Vergara [1975]; Wu et al. [2005]). Moreover, mesocotyl elongation in south and southwest Asian accessions displays a larger variation than that of east Asian ones (Takahashi et al. [1995]). However, this variation has not been elucidated fully with reference to the genetic backgrounds.

Mesocotyl elongation in rice is controlled by several genetic factors and is also affected by environmental factors. Dilday et al. ([1990]) found that mesocotyl elongation could be inherited stably from generation to generation in semi-dwarf rice cultivars. Furthermore, Mgonja et al. ([1994]) reported the partial dominance and preponderance of additive gene effects for mesocotyl elongation using diallel crosses among 6 rice cultivars. Lin et al. ([2006]) showed that mesocotyl elongation in rice was mainly controlled by 2 recessive genes.

The advancement of molecular marker technology has led to the development of genetic maps that make it possible to identify and locate genes or quantitative trait loci (QTLs) controlling quantitative characters. Several studies were conducted to map QTLs for mesocotyl elongation by using various segregating populations from interspecific or intrasubspecific crosses (Cai and Morishima [2002]; Cao et al. [2002]; Katsuta-Seki et al. [1996]; Redoña and Mackill [1996]; Huang et al. [2010]). Five QTLs for mesocotyl length were identified by the slant-board test using an F_2_ population derived from a cross between a low-vigor *japonica* cultivar and a high-vigor *indica* cultivar (Redoña and Mackill [1996]). Eleven QTLs were detected using a recombinant inbred line (RIL) population from an interspecific cross between *O. sativa* and *O. rufipogon* (Cai and Morishima [2002]). Three QTLs controlling mesocotyl length were identified by the glass tube test using an F_2:3_ population of the cross between Assam cultivar “Surjamkhi” and a Chinese *indica* cultivar “Dao Ren Qiao” (Katsuta-Seki et al. [1996]). Cao et al. ([2002]) detected 8 QTLs using a doubled haploid population from *indica* *japonica* cultivar cross. Finally, Huang et al. ([2010]) detected 5 QTLs for mesocotyl length using a RIL population from the cross between 2 *japonica* cultivars under water and plant hormone gibberellins (GA) germination condition. However, the QTLs detected in these studies were not confirmed in a near-isogenic background using chromosome segment substitution lines (CSSLs), and the interaction among the QTLs has not been elucidated.

The aims of this study were 1) to identify QTLs controlling mesocotyl elongation using backcross inbred lines (BILs) derived from a cross between Nipponbare and Kasalath, 2) to confirm and fine-map the QTLs detected in the BILs by using CSSLs and their progeny, and 3) to analyze the interaction of QTLs in controlling mesocotyl elongation.

## Results

### Variation of mesocotyl elongation in back-cross inbred lines (BILs) developed from Kasalath and Nipponbare

Fifty-seven rice accessions from the Rice Diversity Research Set (RDRS) were evaluated for mesocotyl elongation ( Additional file 1: Table S1). Among these accessions, WRC29 (Kalo Dhan) showed the greatest mesocotyl elongation, followed by WRC02 (Kasalath). WRC01 (Nipponbare) was among the accessions showing the shortest mesocotyl length. The mesocotyls of WRC07 (Davao1), WRC20 (Tadukan), and WRC24 (Pinulupot1) did not elongate. Based on the data, the BILs from a cross between Kasalath and Nipponbare were considered suitable for a QTL analysis of mesocotyl elongation.

**Figure 1:**
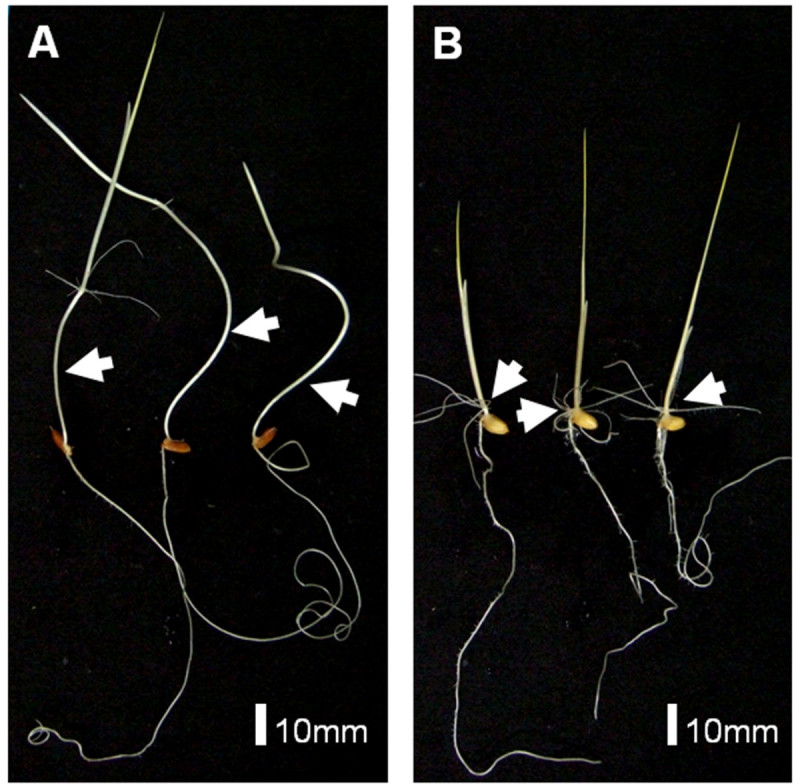
**Seedlings of parental plants, Kasalath (A) and Nipponbare (B), growing for 7days in darkness.** Arrows indicate mesocotyl.

**Figure 2:**
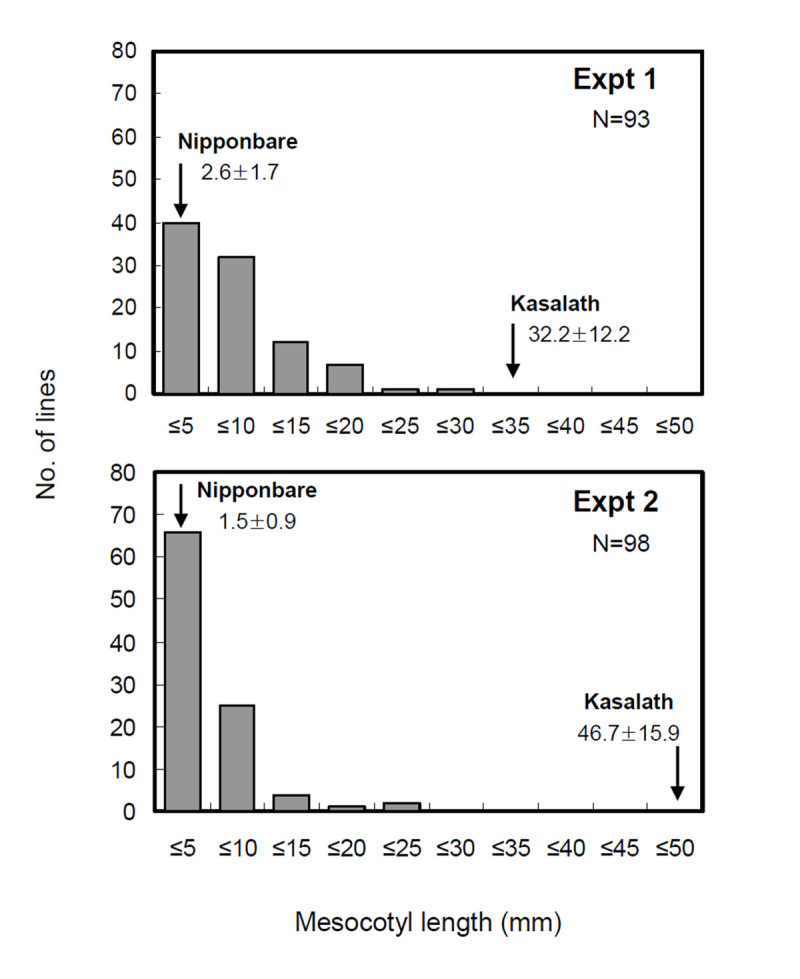
**Frequency distribution of the mesocotyl length of BILs in the two experiments.** Arrow indicates mean with SD for Nipponbare and Kasalath (Expt. 1: n = 30; Expt. 2: n = 36).

Two independent measurements of the mesocotyl length of 98 BILs derived from these accessions were carried out under the agar conditions. A significant difference was found in the mesocotyl length between the 2 parents, Nipponbare and Kasalath (Figures 1 and 2). The mesocotyl of Kasalath elongated over 32.2 mm, whereas that of Nipponbare was less than 2.6 mm. The average mesocotyl length was 7.4 mm and 4.6 mm in the BILs in 2 experiments, while the mesocotyl length of the BILs ranged from 0 to 30 mm. Remarkably, no transgressive segregant with a longer mesocotyl than Kasalath was observed. The distribution of the mesocotyl length between the 2 experiments was somewhat different, although the correlation coefficient was significant (r = 0.86, *P* < 0.001). Kasalath showed more elongation in Expt. 2 (46.7 mm) than Expt. 1 (32.2 mm), and more lines showed reduced mesocotyl elongation in Expt. 2. These results indicate that mesocotyl elongation is influenced by environmental conditions.

### QTLs for mesocotyl elongation

**Figure 3:**
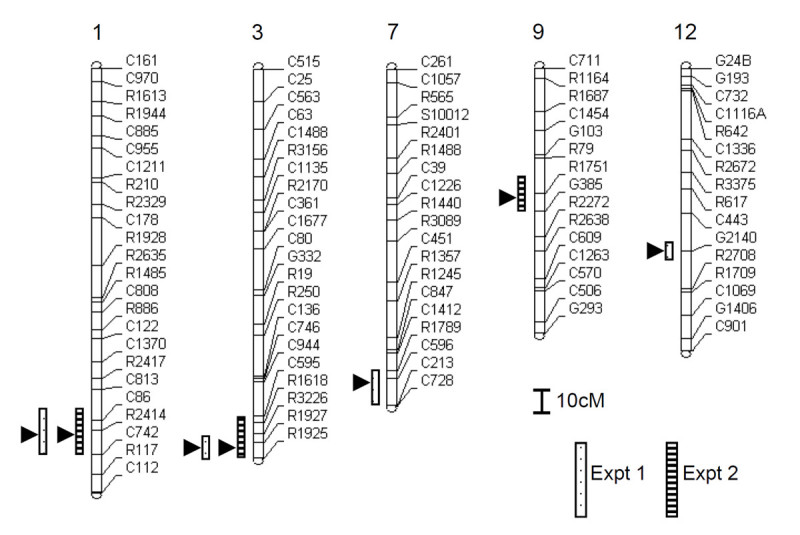
**Chromosomal locations of QTLs for mesocotyl length of BILs in two experiments**. Vertical boxes to the left of each chromosome represent the putative genomic regions harboring QTLs for mesocotyl length (*P* < 0.05). Arrowheads indicate the position with the highest LOD score for each QTL.

A total of 5 QTLs were detected on chromosomes 1, 3, 7, 9, and 12 in the 2 experiments with the BILs (Table 1, Figure 3). When the mean value of two experiments was used in QTL analysis, 4 QTLs were detected in the same locus on chromosomes 1, 3, 7 and 9 except for chromosome 12 (data not shown). QTLs that mapped near the markers R2414 and R1927 on chromosomes 1 and 3, respectively, were detected in both experiments. The *qMel-1* QTL accounted for 15.9% (Expt. 1) and 22.6% (Expt. 2) of the phenotypic variance, whereas *qMel-3* explained 11.5% (Expt. 1) and 20.8% of the variance (Expt. 2). Three additional QTLs, *qMel-7*, *qMel-9*, and *qMel-12*, were each identified in only 1 experiment and accounted for 15.9%, 12.6%, and 9.9% of the phenotypic variance. Kasalath alleles at all QTL loci contributed to an increase in mesocotyl length. The Kasalath alleles at *qMel-1* and *qMel-3* increased the mesocotyl length by 4.4–5.0 mm. Because *qMel-1* and *qMel-3* were detected in both experiments, they were chosen as the targets for fine mapping.

**Table 1:** **Characteristics of QTLs for mesocotyl length in the backcross inbred lines (BILs) in two experiments**

Locus ^a^	Chr.	Marker interval ^b^	Experiment	LOD score ^c^	R^*2*^ (%) ^d^	Additive effect ^e^
*qMel-1*	1	C86/R2414/C742	Expt 1	5.4	15.9	5.0
		C86/R2414/C742	Expt 2	8.3	22.6	4.6
*qMel-3*	3	R3226/R1927/R1925	Expt 1	4.2	11.5	4.4
		C595/R1927/R1925	Expt 2	7.5	20.8	4.5
*qMel-7*	7	R1789/C596/C213	Expt 1	6.0	15.9	4.8
*qMel-9*	9	R79/R1751/G385	Expt 2	5.1	12.6	3.6
*qMel-12*	12	C443/G2140/R2708	Expt 1	3.2	9.9	4.6

^a^ QTLs were designated as “*qMel*-chromosome number”

^b^ The nearest RFLP marker to the QTL is underlined

^c^ Putative QTLs with significant LOD score tested at *P* < 0.05

^d^ Proportion of the phenotypic variance explained by the nearest marker of QTL

^e^ Estimated effect of replacing Nipponbare alleles by Kasalath alleles

**Figure 4:**
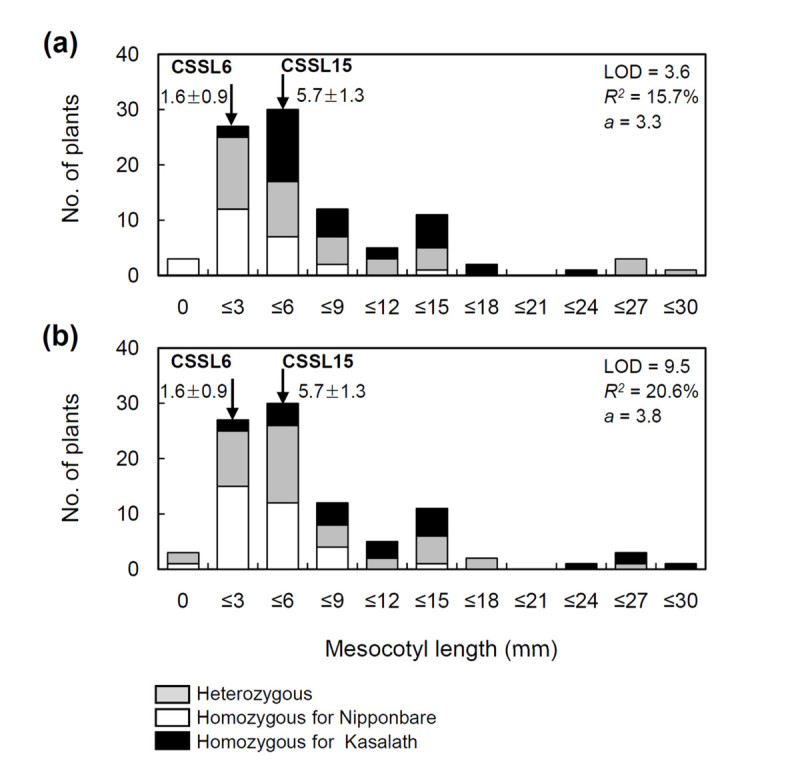
**Frequency distribution of the mesocotyl length of 95 F**_**2**_ **plants derived from a cross between CSSL6 and CSSL15.** Arrows indicate mean values with standard deviations (n = 11) for CSSL6 and CSSL15. White, black and gray bars indicate homozygous for Nipponbare and Kasalath alleles and heterozygous for the marker RM3602 on chromosome 1 (**a**) and RM8277 on chromosome 3 (**b**), respectively. The LOD score (LOD), proportion of the phenotypic variance (*R*^*2*^) and the additive effect of the Kasalath allele (*a*) are indicated in each figure (*P* < 0.05).

For confirming these 2 QTLs, CSSL-6 and CSSL-15, carrying the QTLs *qMel-1* and *qMel-3*, respectively, were crossed to develop an F_2_ population. A total of 95 F_2_ plants were measured for mesocotyl length, and then *qMel-1* and *qMel-3* were mapped with 10 SSR markers. Figure 4 shows the distribution of mesocotyl length based on the genotype of the nearest SSR markers, RM3602 at the *qMel-1* region and RM8277 at the *qMel-3* region, in the F_2_ population. RM3602 linked to *qMel-1* explained 15.7% of the phenotypic variance, whereas RM8277 on chromosome 3 accounted for 20.6% of the variance in the 95 F_2_ population. The mesocotyls of CSSL-6 and CSSL-15 were 1.6 and 5.7 mm, respectively, and those of the F_2_ population ranged from 0 to 30 mm. Transgressive segregants exceeding the parental values were also observed. The distribution showed continuous but not a normal distribution. Therefore, clearly classifying the F_2_ plants into subgroups according to mesocotyl length by the genotype data was impractical.

### Interaction between *qMel-1* and *qMel-3*

**Figure 5:**
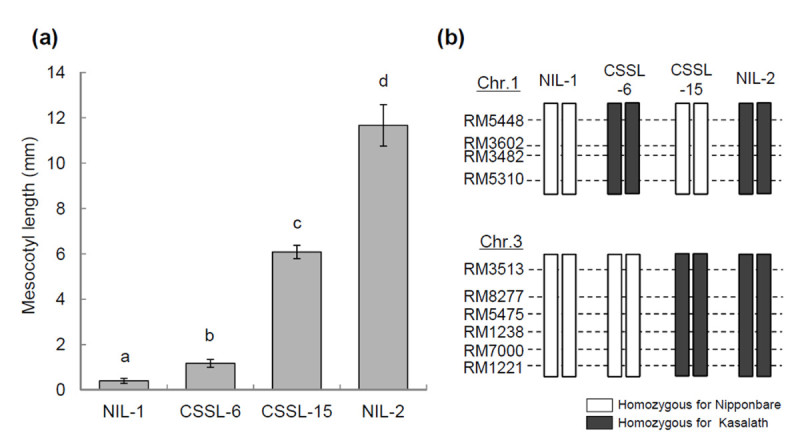
**Comparison of mesocotyl length in 2 NILs and 2 parental lines, CSSL-6 and CSSL-15 with different genotypes at** ***qMel-1*** **and** ***qMel-3*** **.** (**a**) Mean mesocotyl length with SE of 4 lines. (**b**) Graphical representation of the genotypes of 4 lines. Pair-wise comparison was conducted between each line based on the Duncan’s multiple range test. Means with the different letter are significantly different at P = 0.001.

To test the interaction between the 2 QTLs, we measured the mesocotyl length of 4 lines, NIL-1, CSSL-6, CSSL-15, and NIL-2 (Figure 5). Mesocotyl of the CSSL-6 was significantly longer than that of NIL-1 (*P* < 0.001). The mesocotyls of CSSL-15 plants were significantly increased in length relative to those from CSSL-6 (*P* < 0.001) and NIL-1 plants (*P* < 0.0001). In addition, the mesocotyls of NIL-2 plants were significantly longer than those of CSSL-15 plants (*P* < 0.00001). In the F_2_ population, two-way ANOVA revealed a non-significant digenic interaction between two markers, RM3602 and RM8277 linked to *qMel-1* and *qMel-3*, respectively (*P* = 0.31) (data not shown). These results indicate that the 2 QTLs act additively in distinct or complementary pathways in controlling mesocotyl elongation.

### Substitution mapping

**Figure 6:**
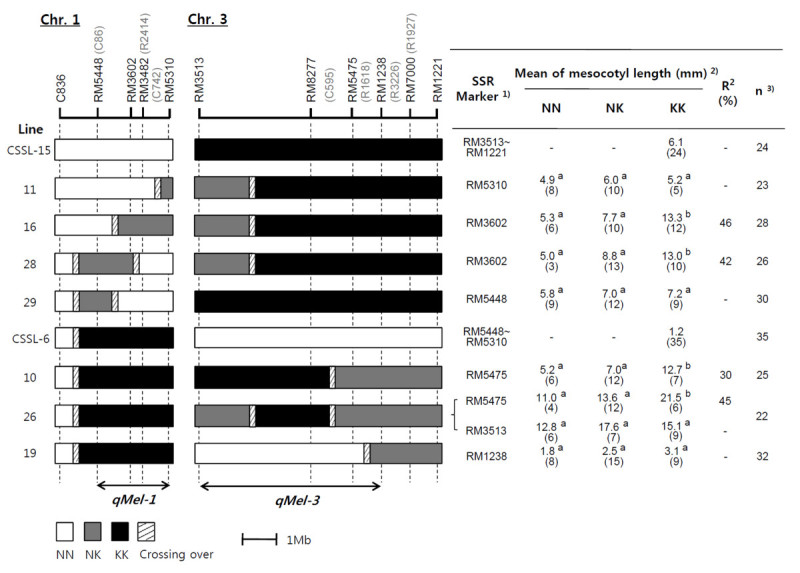
**Graphical genotypes of F**_**3**_**lines used in substitution mapping of** ***qMel-1*** **and** ***qMel-3***. White portions of the graph indicate homozygous Nipponbare chromosome segments, black regions indicate homozygous Kasalath chromosomes, gray areas indicate heterozygous regions and slashed areas are regions where crossing-over occurred. The table to the right of the graphical genotypes indicates mean mesocotyl length for each of the three genotypes of F_3_ lines and two CSSLs. One line was genotyped with two markers, RM5475 and RM3513. The broken vertical lines define the interval containing the *qMel-1* and *qMel-3* loci. 1) Markers within the heterozygous regions were tested and the ones with the highest R^2^ scores are shown. 2) Numbers followed by the different letter in each row are significantly different at *P* = 0.05 based on the Duncan’s multiple range test. NN: Nipponbare homozygotes, NK: Nipponbare/Kasalath heterozygotes, KK: Kasalath homozygotes, 3) n: number of evaluated individuals in each line. * Number in () indicate the number of F_3_ plants in each genotype.

Based on the finding that the 2 QTLs behave in a complementary manner, 4 and 3 F_2_ plants with informative recombination breakpoints within the *qMel-1* and *qMel-3* regions of the introgressed Kasalath segments, respectively, were identified and selfed to produce F_3_ lines for substitution mapping (Figure 6). Four lines (lines 11, 16, 28, and 29) were homozygous for Kasalath across the *qMel-3* region defined by SSR markers RM8277 and RM1221; 3 lines (lines 10, 26, and 19) were homozygous at the *qMel-1* region defined by SSR markers RM5448 and RM5310. The F_3_ lines were used to explore the dominance relationship among alleles at the *qMel-1* and *qMel-3* QTLs. For this purpose, the phenotypic means were compared among the 3 genotypes defined by the allele constitution at RM5448, RM3602 and RM5310 on chromosome 1 and RM3513, RM5475 and RM1238 on chromosome 3 (Figure 6). The mean mesocotyl length of the Nipponbare homozygotes was not significantly different from that of the heterozygotes. However, the mean mesocotyl length of the Kasalath homozygotes at RM3602 and RM5475 was significantly higher than that of the Nipponbare homozygotes and heterozygotes. These results showed that the Nipponbare alleles at the *qMel-1* and *qMel-3* loci were dominant over the Kasalath alleles. The implication from this analysis was that the Nipponbare homozygotes and heterozygotes could be treated together in the phenotypic analysis for the fine mapping of the 2 QTL.

CSSL-6 and CSSL-15 served as respective positive controls for the region as a whole. The mesocotyl lengths of lines 11 and 29 were not significantly different than that of CSSL-15. In addition, the 3 genotypes of the two lines, 11 and 29, did not show significant differences, suggesting that the 2 lines did not contain a Kasalath allele affecting mesocotyl elongation in the introgressed segments. A comparison of mesocotyl length among the F_3_ progeny showed significant differences among the 3 genotypes for the populations from lines 16 and 28. Based on the size of the chromosome 1 introgression in 16 and 28, it was concluded that *qMel-1* was located in the interval RM5448–RM5310, a region of approximately 3,799 kb (Figure 6) (http://www.gramene.org/marker/, Reference to Gramene Annotated Nipponbare Sequence 2009). RM5448 and RM5310 represented the outside borders of the introgression. Hence, we conclude that the *qMel-1* locus has been localized to a region <3,799 kb in size.

For *qMel-3*, the mesocotyl lengths of lines 10 and 26 were significantly different than that of CSSL-6, and the 3 genotypic classes for lines 10 and 26 showed significant differences at RM5475 but not for line 26 at RM3513. These results suggested that the 2 lines did contain a Kasalath allele affecting mesocotyl elongation in the introgressed Kasalath segment. *qMel-3* was consistently mapped nearest to marker R1927, with this marker explaining 11.5–20.8% of the phenotypic variation of mesocotyl length in 98 BILs. Based on the size of the chromosome 3 introgression in lines 10, 26, and 19, *qMel-3* was concluded to be located in the interval RM3513–RM1238, a region of ~6,964-kb (Figure 6) (http://www.gramene.org/marker/, Reference to Gramene Annotated Nipponbare Sequence 2009).

## Discussion

This study was conducted to identify QTLs controlling mesocotyl elongation in rice. Mesocotyl length is a quantitative trait and displays a substantial amount of variation among genotypes, especially in *indica* cultivars (Takahashi et al. 1978; Takahashi et al. [1995]; Wu et al. [2005]). Phenotyping of 57 rice accessions provided valuable information about the range and distribution of mesocotyl length in rice. The mean mesocotyl length in *indica* was nearly twice that of *japonica*, and *indica* accessions showed a larger variation than *japonica* accessions in mesocotyl length. The mesocotyl length of *indica* accessions ranged from 0 mm to 46.0 mm, whereas that of *japonica* cultivars ranged from 0 mm to 16.4 mm on agar medium (Additional file 1: Table S1). This result suggests that *indica* germplasms contain alleles that would be useful sources of genetic variation for enhancing mesocotyl elongation in *japonica*.

Mesocotyl elongation is sensitive to environmental factors. Mesocotyl growth is affected by light (Takahashi [1984]; Nick and Furuya [1993]), moisture (Takahashi [1978]), and temperature. It is noteworthy that no difference in R^2^ values for *qMel-1* and *qMel-3* between the 2 mapping populations was observed. R^2^ values for *qMel-1* were 15.9% and 22.6% in the BILs and 15.7% in the F_2_ population (Table 1 and Figure 4). This result is not consistent with those of previous studies showing that the proportion of the phenotypic variance that could be explained by the markers was greatly enhanced in the NIL population compared with segregating populations, such as BILs. This is mainly due to the fact that mesocotyl elongation is affected by environmental conditions and that a single plant was measured for mesocotyl elongation in the F_2_ population. Multiple regression analysis in the BIL population also indicated that five QTL explained only 54.0% of the total phenotypic variance. It is interesting that CSSL-6 showed contrasting results in mesocotyl length according to experiments. Mesocotyl of CSSL-6 was significantly longer than that of NIL-1 in the experiment to detect interaction between 2 QTLs (Figure 5). However, no difference in mesocotyl length between Nipponbare and CSSL-6 was not observed in the substitution mapping experiment, although the mesocotyls of CSSL-6 plants were longer than those of Nipponbare (data not shown). These results are similar to the complementary effect of two genes, *Rc* and *Rd* in coloration in rice grains except that each of these two genes is inherited monogenetically whereas *qMel-1* and *qMel-3* show quantitative inheritance (Furukawa et al. [2006]). In red coloration in rice grains, the *Rc* and *Rd* genes are necessary for the red pigmentation, *Rc* and *rd* are involved in the brown pigmentation, and either *rc* and *rd* or *rc* and *Rd* produce white grains. These results might suggest that *qMel-1* requires the complementary effects of other QTL in mesocotyl elongation or *qMel-1* is more sensitive to environment conditions.

A number of studies reported various QTLs for mesocotyl elongation using interspecific and intersubspecific crosses (Cai and Morishima [2002]; Cao et al. [2002]; Katsuta-Seki et al. [1996]; Redoña and Mackill [1996]; Huang et al. [2010]). Eleven QTLs for mesocotyl elongation were identified on chromosomes 1, 3, 4, 5, 6, 9 and 11 using an RIL population derived from a cross between an *indica* cultivar and wild rice, *O. rufipogon* (Cai and Morishima [2002]). Cao et al. ([2002]) detected eight QTLs on chromosomes 1, 3, 6, 7, 8, and 12 using a doubled haploid population from a cross between IR64 and Azucena. Five QTLs for mesocotyl elongation were mapped on chromosomes 1, 3, 5 and 7 using an F_3_ population developed from a cross between *japonica* cultivar, Labelle and *indica* cultivar, Black Gora (Redoña and Mackill [1996]). Of interest, QTLs for mesocotyl elongation were commonly mapped to chromosomes 1 and 3 in different mapping populations and experiment conditions. We also identified QTLs *qMel-1* and *qMel-3* on chromosomes 1 and 3, respectively, in both experiments in this study (Figure 6). These 2 QTLs detected in the present study are located in an interval similar to ones reported by other previous studies. *qMel-1* was colocalized with QTLs for mesocotyl elongation in previous reports (Cai and Morishima [2002]; Cao et al. [2002]; Katsuta-Seki et al. [1996]; Redoña and Mackill [1996]). *qMel-3* was located in the RM3513-RM1238 interval on the long arm of chromosome 3, and this interval overlapped with regions of QTLs reported in previous studies (Cai and Morishima [2002]; Cao et al. [2002]; Katsuta-Seki et al. [1996]; Redoña and Mackill [1996]; Huang et al. [2010]). These results clearly demonstrated the existence of QTLs controlling mesocotyl elongation on chromosomes 1 and 3. Previous studies identified a QTL for mesocotyl length in a putatively homeologous location on the long arm of maize chomosome 3 (Troyer [1997]; Zhang et al. [2012]). Troyer ([1997]) found three regions on chromosome 3, 6 and 9 related to mesocotyl elongation using translocation tester stocks. Three QTLs for mesocotyl length were commonly detected on chromosome 1, 3 and 10 under two different sowing depth conditions in maize (Zhang et al. [2012]). While the resolution of the maize QTL was low, the positional correspondence raises the possibility that this locus may be involved in controlling mesocotyl length in both rice and maize (Wei et al. [2007]; Soderlund et al. [2011]http://www.symapdb.org).

The Kasalath alleles at *qMel-1* and *qMel-3* increased mesocotyl length in the isogenic Nipponbare background. These results indicate that the 2 QTLs act additively in complementary pathways in controlling mesocotyl elongation. To the best of our knowledge, this is the first study using CSSLs to reveal a complementary effect between QTLs for mesocotyl length.

Substitution mapping has been applied in diverse plant species to facilitate the fine mapping of QTLs (Wissuwa et al. [2002]; Li et al. [2004]). Based on substitution mapping, *qMel-1* was mapped to a 3,799-kb interval between markers RM5448 and RM5310 on chromosome 1, while *qMel-3* was mapped to a 6,964-kb interval between markers RM3513 and RM1238 on chromosome 3. Based on the annotated Nipponbare genome sequence, the 3,799-kb interval with the *qMel-1* locus and 6,964-kb interval with the *qMel-3* contain 490 and 700 putative genes, respectively (http://www.ncbi.nlm.nih.gov/projects/mapview/map_search.cgi?taxid=4530&query=).

## Conclusions

Our analysis of the *qMel-1* and *qMel-3* locus led to the delimitation of regions of chromosomes 1 and 3, as well as the development of several molecular markers suitable for marker-aided selection for mesocotyl length. Fine-mapping of these loci along with sequence and expression analysis, is underway to clone the genes using a map-based cloning strategy. The NIL populations and molecular markers are useful materials for the cloning of these QTLs. To date, a number of QTLs for mesocotyl elongation have been identified using a variety of cross combinations in rice (http://www.gramene.org). Overall, our analysis indicated that more comprehensive research on epistasis among QTLs is necessary to provide enough data to facilitate the accumulation of desirable QTLs in breeding lines and to better understand the genetic mechanism controlling mesocotyl elongation.

## Methods

### Plant materials

Fifty-seven rice accessions selected from the Rice Diversity Research Set (RDRS) of germplasm collection were used to detect variation in mesocotyl elongation in cultivated rice (*Oryza sativa* L.) (Kojima et al. [2005]; Additional file 1: Table S1). The original seeds of the RDRS collection were provided by the Genebank of the National Institute of Agrobiological Sciences, Japan (http://www.gene.affrc.go.jp/databases-core_collections_wr_en.php).

To identify QTLs for mesocotyl elongation, we used 98 BILs that had been developed from BC_1_F_1_ of the Nipponbare/Kasalath//Nipponbare cross by the single-seed descent method (Lin et al. [1998]).

For confirmation and fine mapping of *qMel-1* and *qMel-3*, 2 CSSLs, CSSL-6 and CSSL-15 were selected from the CSSLs developed from a cross between Nipponbare and Kasalath at Rice Genome Resource Center (RGRC), Japan (http://www.rgrc.dna.affrc.go.jp/ineNKCSSL54.html). CSSL-6 and CSSL-15, carrying the QTLs *qMel-1* and *qMel-3*, respectively, were crossed to develop an F_2:3_ population. CSSL-6 contained 2 Kasalath introgressions, a 34.7 cM-sized segment from the end of the long arm to restriction fragment length polymorphism (RFLP) marker C86 of chromosome 1 and a 6.3 cM segment flanked by RFLP markers, C39-R1440 on chromosome 7, in the Nipponbare background. The Kasalath segment on chromosome 1 of CSSL-6 included *qMel-1*. In CSSL-15, a 61.3 cM-sized Kasalath segment was introgressed near RFLP markers, R19-R1925 on the long arm of chromosome 3. The resulting 3 F_1_ plants were selfed to obtain F_2_ plants. Ninety-five F_2_ plants were generated and used to confirm the target QTLs. Thirty-two F_2_ plants with recombination breakpoints within the target QTL regions were selected and selfed to obtain F_3_ seeds for substitution mapping.

Seeds of BILs and CSSLs, which were derived from a cross between Nipponbare and Kasalath were provided by RGRC, Japan (http://www.rgrc.dna.affrc.go.jp/stock.html). RDRS and BILs plants were grown in the experimental lowland field of the Graduate School of Life Sciences, Tohoku University at Kashimadai, Osaki, Miyagi Prefecture, Japan. After the mesocotyl lengths of the F_2_ and F_3_ populations from a cross between CSSL-6 and CSSL-15 were measured, the individual plants were planted in the greenhouse. The panicles of these plants were harvested at 40–50 days after heading and then dried in a well-ventilated room for 3 months. Seeds were removed from the dried panicles by hand, and then only seeds without any visible damage were selected for this study. The seeds were placed in paper envelopes and packed in plastic sealed bags with silica gel. The seeds were then stored in the 4°C refrigerator until the experiments.

### Evaluation of mesocotyl elongation

To measure mesocotyl elongation, we used plastic jars for plant culture (70-mm diameter, 120-mm height; Sansyo Ltd., Tokyo, Japan) containing 30 ml of 0.3% agar medium. Twelve good quality seeds from each RDRS accession, 98 BILs, F_2_ and F_3_ population were sown at a 1 cm depth in the agar medium of each jar in 2 replications and immediately placed into a black box (60 cm × 44 cm, 29 cm height) ventilated by an air-pump (1 lit./min) in a 30°C dark room. At 7 day after the start of incubation, the mesocotyl length of each seedling was measured by a ruler as the distance from the basal part of the seminal root to the coleoptilar node. Seedlings that grew poorly were excluded from the measurements for mesocotyl length. The means in each temporal replication (Expt. 1 and Expt. 2) were used for the QTL analysis.

### QTL analysis in the BIL and F_2_ populations

To map QTLs for mesocotyl elongation in the BILs, the genotype data generated using 245 RFLP markers (http://rgp.dna.affrc.go.jp/publicdata/genotypedataBILs/genotypedata.html) were used. For detecting the precise location of the target QTL, a total of 95 F_2_ plants were subjected to linkage analysis with 4 and 6 SSR markers on the target regions of chromosomes 1 and 3, respectively (McCouch et al. [2002]).

Linkage analysis was performed using the Kosambi function of Mapmaker/EXP 3.0 software (Lander et al. [1987]). QTL analysis was performed by composite interval mapping (CIM) using the QTL Cartographer version 2.5 software (Wang et al. [2007]). CIM analysis was performed with a forward-backward stepwise regression-using model 6 with a 10 cM window size. The log-likelihood (LOD) threshold significance level (*P* < 0.05) was determined by computing 1,000 permutations. The QTL positions were assigned to the point of the maximum LOD score in the target regions. The percentage of the total phenotypic variance accounted for by each QTL was estimated on the basis of the R^2^ value.

### Substitution mapping of *qMel-1* and *qMel-3* and interaction analysis

Seven F_2_ plants with different recombination breakpoints across the *qMel-1* or *qMel-3* regions were identified and selfed to develop F_3_ lines from 32 F_2_ plants. After the mesocotyl length of each F_3_ plant from each line was measured, the seedlings were grown in a glass test tube for DNA extraction and genotyping with SSR markers for substitution mapping of the target QTL.

Two F_2_ plants were identified with the following genotypes: NIL-1 (Nipponbare homozygous at both QTL loci) and NIL-2 (Kasalath homozygous at both QTL loci). A single F_2_ plant per each NIL group was selfed to produce an F_3_ line, and these 2 F_3_ lines, in addition to CSSL-6 and CSSL-15, were evaluated for mesocotyl length to test the interaction between the 2 QTLs.

### DNA marker analysis

DNA was extracted from BILs in bulk and from each of the F_2_ and F_3_ plants derived from the CSSL-6 x CSSL-15 cross. A piece of leaf, 5 cm in length, was cut from the tip of each leaf blade, placed in a microtube containing extraction buffer [200 mM Tris-HCl (pH 8.0), 250 mM NaCl, 25 mM EDTA, 0.5% (w/v) sodium dodecyl sulfate], and homogenized with a pestle. DNA was precipitated with isopropanol and then resuspended in 50ul of TE buffer [10 mM Tris-HCl (pH 8.0), 1 mM EDTA]. PCR amplification fragments were separated using electrophoresis on a 2.7% agarose gel.

## Electronic supplementary material

Additional file 1: Table S1: Variation of the mesocotyl length for 57 rice accessions from RDRS collection. (PDF 114 KB)

Below are the links to the authors’ original submitted files for images.

Authors’ original file for figure 1

Authors’ original file for figure 2

Authors’ original file for figure 3

Authors’ original file for figure 4

Authors’ original file for figure 5

Authors’ original file for figure 6
