# Supplementary material for: Mapping and characterization of quantitative trait loci for mesocotyl elongation in rice (Oryza sativa L.)
Source: Rice (N Y). 2012 Jun 26;5:13. doi: 10.1186/1939-8433-5-13 (PMC5520832; doi:10.1186/1939-8433-5-13)
Supplement: Supplementary file 1 — Additional file 1: Table S1: Variation of the mesocotyl length for 57 rice accessions from RDRS collection. (PDF 114 KB) [file 12284_2012_12_MOESM1_ESM.pdf]

Additional table 1. Variation of the mesocotyl length for 57 rice accessions from RDRS collection

No.	**Name**	**WRC No.**[a]	**Origin**	**Variety group**	**Mesocotyl length (mm)**		
					**Mean**		**S.D**
1	NIPPONBARE	WRC 01	JAPAN	*Japonica*	1.7	±	0.4
2	KASALATH	WRC 02	INDIA	*Indica*	41.9	±	2.3
3	BEI KHE	WRC 03	CAMBODIA	*Indica*	3.1	±	0.4
4	JENA 035	WRC 04	NEPAL	*Indica*	21.6	±	3.1
5	NABA	WRC 05	INDIA	*Indica*	12.7	±	0.8
6	PULUIK ARANG	WRC 06	INDONESIA	*Indica*	9.5	±	1.0
7	DAVAO 1	WRC 07	PHILIPPINES	*Indica*	0.0	±	0.0
8	RYOU SUISAN KOUMAI	WRC 09	CHINA	*Indica*	3.3	±	0.4
9	SHUUSOUSHU	WRC 10	CHINA	*Indica*	14.8	±	1.6
10	JINGUOYIN	WRC 11	CHINA	*Indica*	1.3	±	0.2
11	ASU	WRC 13	BHUTAN	*Indica*	9.5	±	0.7
12	IR 58	WRC 14	PHILIPPINES	*Indica*	7.0	±	0.8
13	CO 13	WRC 15	INDIA	*Indica*	2.3	±	0.5
14	KEIBOBA	WRC 17	CHINA	*Indica*	10.8	±	1.4
15	QINGYU(SEIYU)	WRC 18	TAIWAN	*Indica*	3.7	±	0.7
16	DENG PAO ZHAI	WRC 19	CHINA	*Indica*	2.7	±	0.4
17	TADUKAN	WRC 20	PHILIPPINES	*Indica*	0.0	±	0.0
18	SHWE NANG GYI	WRC 21	MYANMAR(BURMA)	*Indica*	1.8	±	0.2
19	CALOTOC	WRC 22	PHILIPPINES	-	21.0	±	1.5
20	LEBED	WRC 23	PHILIPPINES	-	14.8	±	1.5
21	PINULUPOT 1	WRC 24	PHILIPPINES	-	0.0	±	0.0
22	MUHA	WRC 25	INDIA	*Indica*	25.3	±	1.6
23	JHONA 2	WRC 26	INDIA	*Indica*	9.4	±	0.9
24	NEPAL 8	WRC 27	NEPAL	*Indica*	17.4	±	0.9
25	JARJAN	WRC 28	BHUTAN	*Indica*	19.8	±	2.7
26	KALO DHAN	WRC 29	NEPAL	*Indica*	46.0	±	3.1
27	ANJANA DHAN	WRC 30	NEPAL	*Indica*	19.6	±	2.1
28	SHONI	WRC 31	BANGLADESH	*Indica*	5.6	±	1.4

29	TUPA 121-3	WRC 32	BANGLADESH	*Indica*	33.1	±	4.2
30	SURJAMUKHI	WRC 33	INDIA	*Indica*	7.5	±	1.0
31	ARC 7291	WRC 34	INDIA	*Indica*	10.9	±	1.6
32	ARC 5955	WRC 35	INDIA	*Indica*	15.3	±	0.7
33	RATUL	WRC 36	INDIA	*Indica*	19.0	±	2.1
34	ARC 7047	WRC 37	INDIA	*Indica*	27.9	±	2.7
35	ARC 11094	WRC 38	INDIA	*Indica*	14.4	±	2.0
36	BADARI DHAN	WRC 39	NEPAL	*Indica*	33.1	±	1.9
37	NEPAL 555	WRC 40	INDIA	*Indica*	35.0	±	3.5
38	KALUHEENATI	WRC 41	SRILANKA	*Indica*	1.8	±	0.3
39	LOCAL BASMATI	WRC 42	INDIA	*Indica*	40.0	±	2.8
40	DIANYU 1	WRC 43	CHINA	*Japonica*	0.9	±	0.4
41	BASILANON	WRC 44	PHILIPPINES	*Indica*	34.8	±	1.5
42	MA SHO	WRC 45	MYANMAR(BURMA)	*Japonica*	13.0	±	1.3
43	KHAO NOK	WRC 46	LAOS	*Japonica*	11.8	±	1.3
44	JAGUARY	WRC 47	BRAZIL	*Japonica*	4.4	±	0.5
45	KHAU MAC KHO	WRC 48	VIETNAM	*Japonica*	11.7	±	1.5
46	PADI PERAK	WRC 49	INDONESIA	*Japonica*	14.1	±	0.8
47	REXMONT	WRC 50	USA	*Japonica*	2.1	±	0.4
48	URASAN 1	WRC 51	JAPAN	*Japonica*	10.6	±	0.8
49	KHAU TAN CHIEM	WRC 52	VIETNAM	*Japonica*	3.0	±	0.3
50	TIMA	WRC 53	BHUTAN	*Japonica*	16.4	±	1.8
51	TUPA 729	WRC 55	BANGLADESH	*Japonica*	9.9	±	0.5
52	MILYANG 23	WRC 57	REP.KOREA	*Indica*	11.0	±	0.4
53	RADIN GOI SESAT	WRC 61	MALAYSIA	*Indica*	3.7	±	0.8
54	BLEIYO	WRC 63	THAILAND	*Indica*	21.7	±	2.4
55	RAMBHOG	WRC 65	INDONESIA	*Indica*	8.4	±	1.6
56	PHULBA	WRC 67	INDIA	*Japonica*	7.3	±	1.7
57	KHAO NAM JEN	WRC 68	LAOS	*Japonica*	4.8	±	1.0

[a] World rice collection (WRC) number corresponds to the accession number of RDRS at the NIAS Gene bank (Kojima et al. 2005).
