# Supplementary figures and images for: Mapping and characterization of quantitative trait loci for mesocotyl elongation in rice (Oryza sativa L.)
Source: Rice (N Y). 2012 Jun 26;5:13. doi: 10.1186/1939-8433-5-13 (PMC5520832; doi:10.1186/1939-8433-5-13)

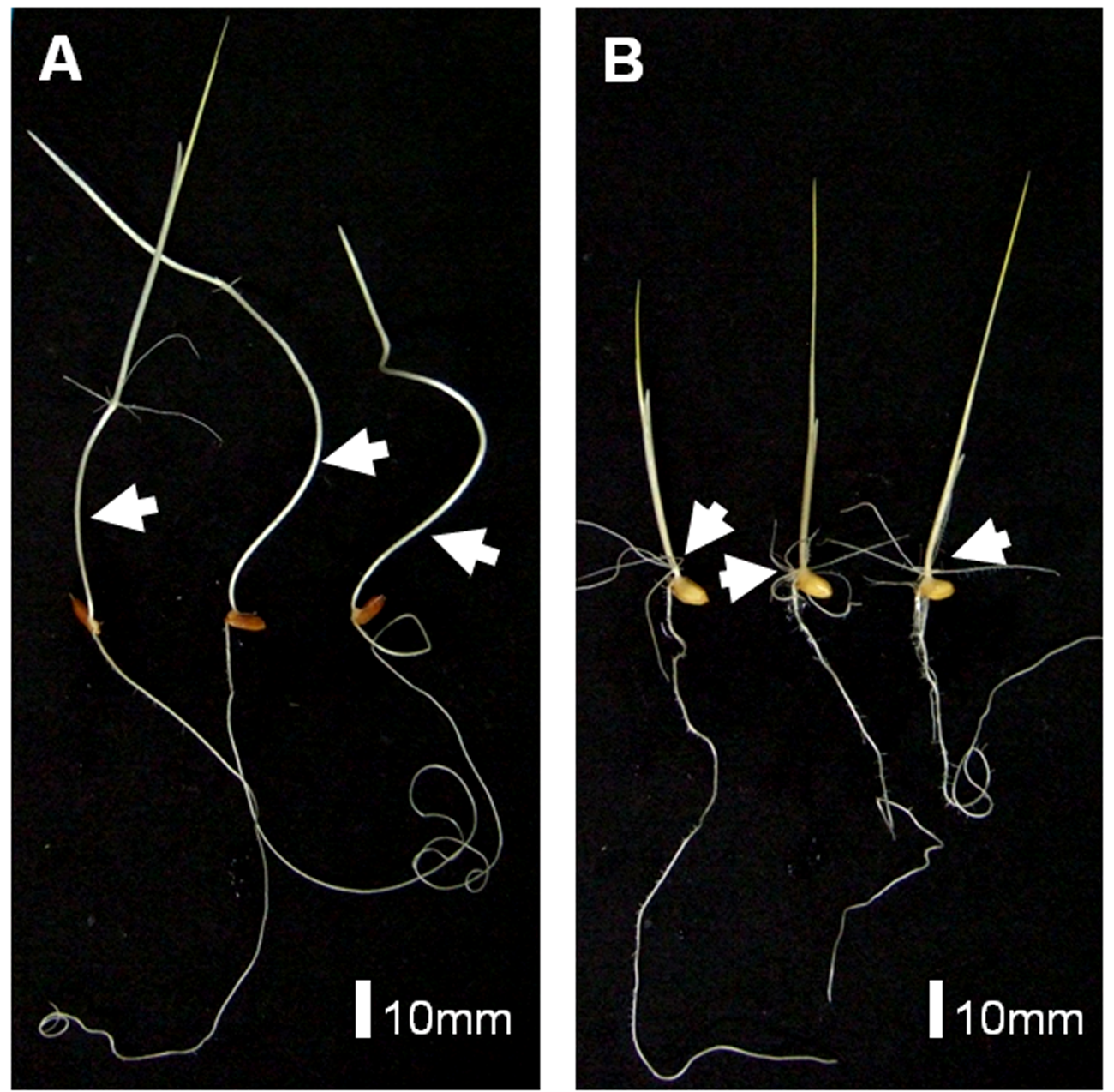

Supplement: Supplementary file 2 — Authors’ original file for figure 1 [file 12284_2012_12_MOESM2_ESM.tiff]

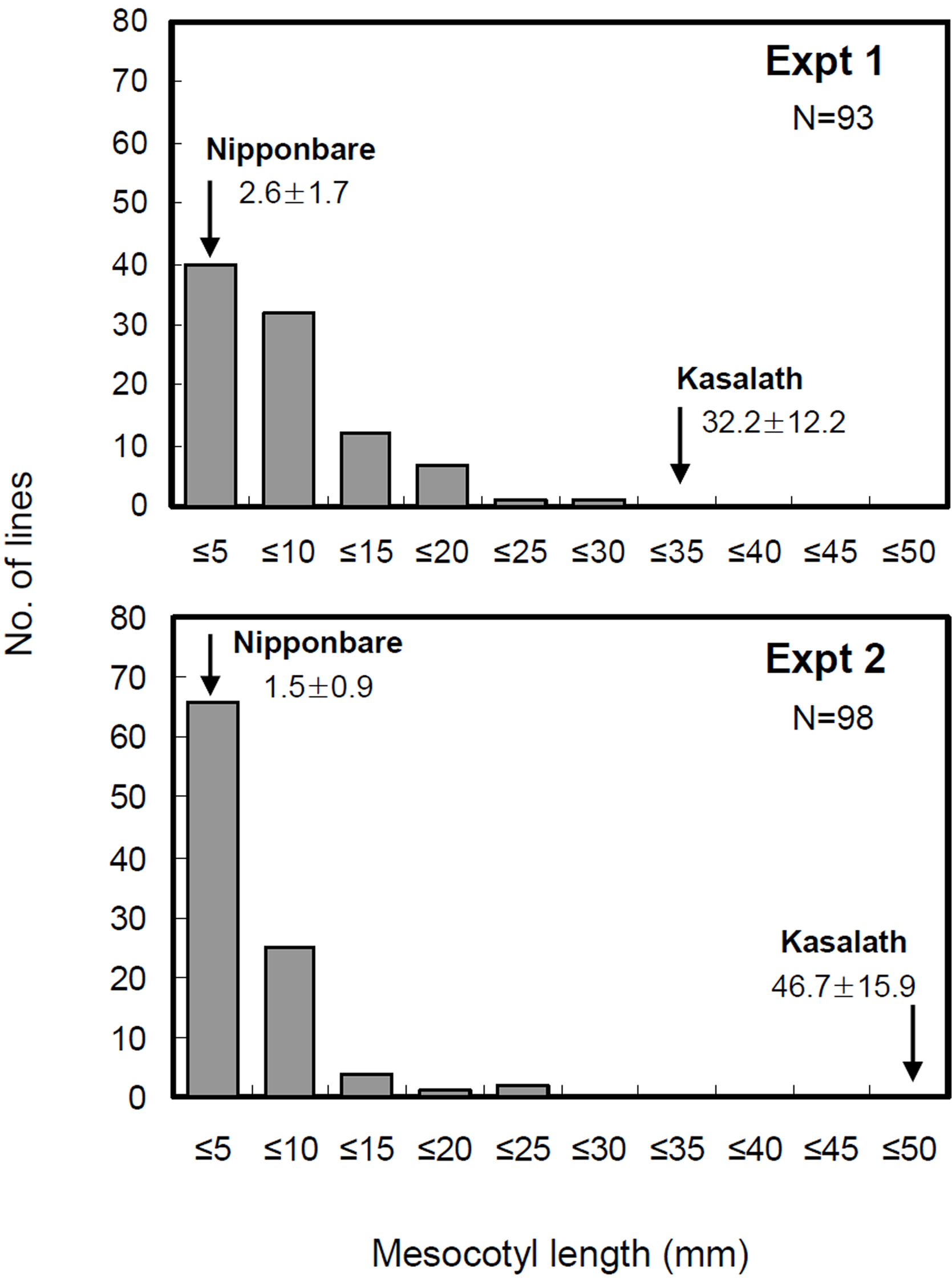

Supplement: Supplementary file 3 — Authors’ original file for figure 2 [file 12284_2012_12_MOESM3_ESM.tiff]

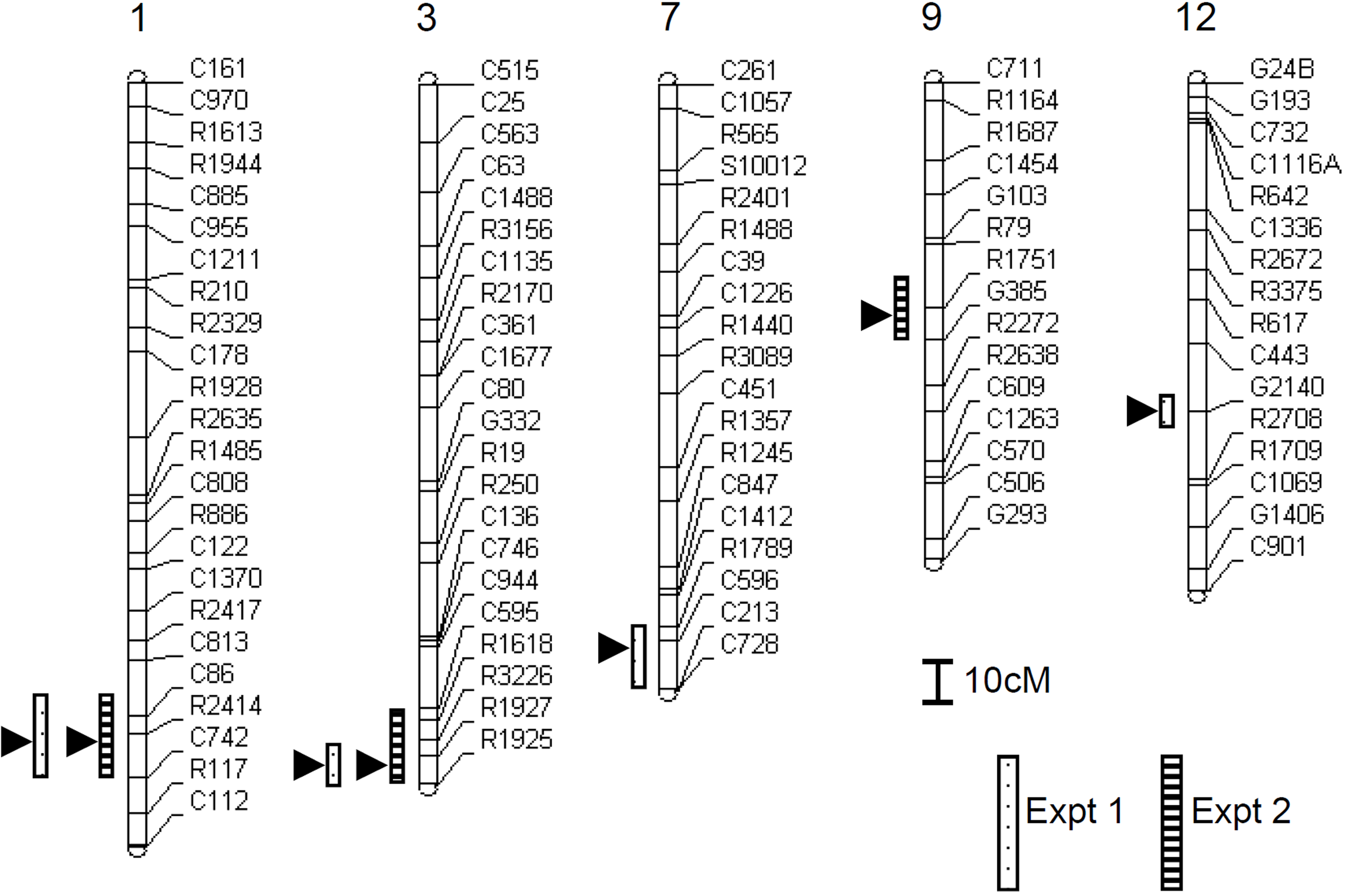

Supplement: Supplementary file 4 — Authors’ original file for figure 3 [file 12284_2012_12_MOESM4_ESM.tiff]

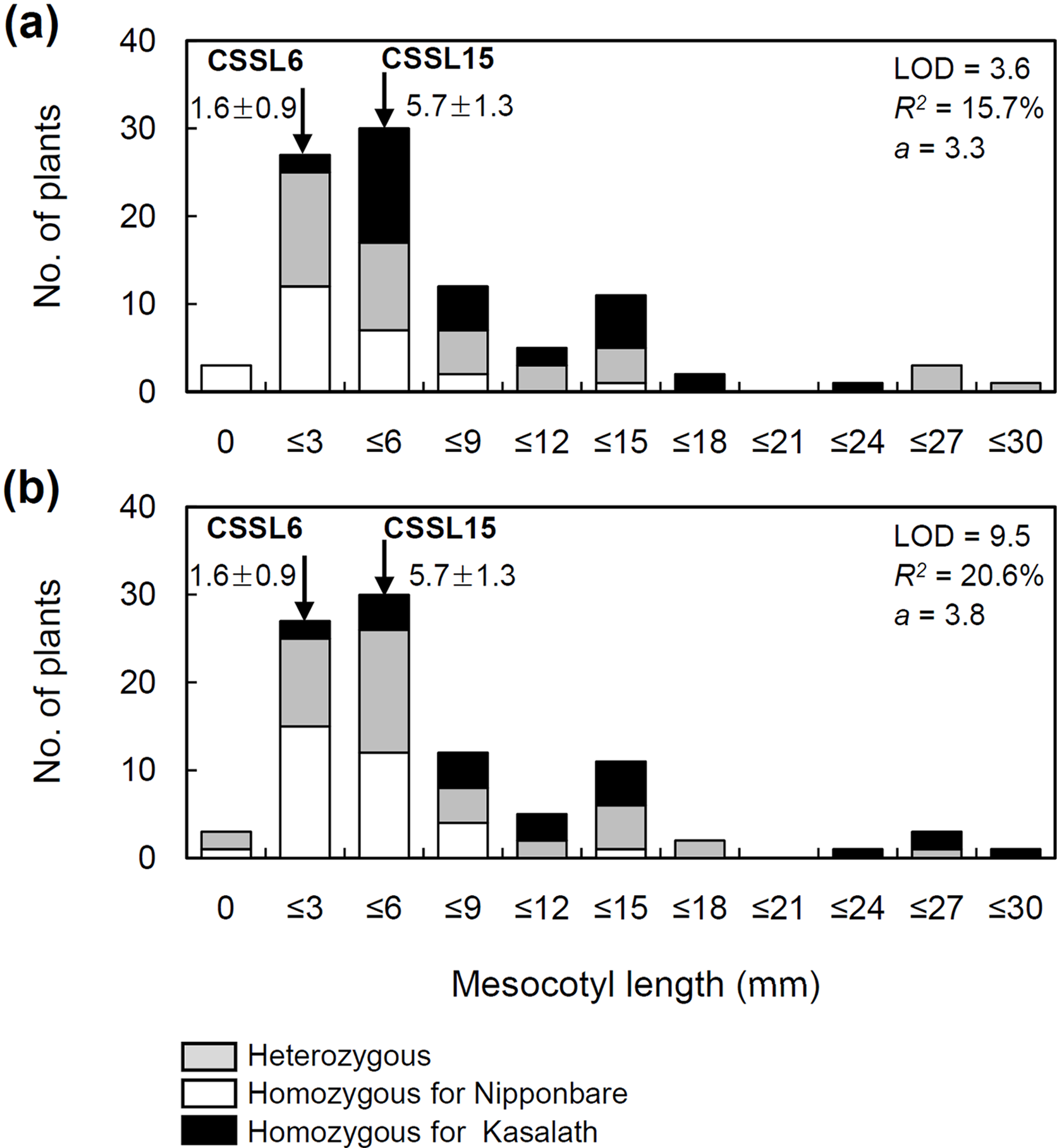

Supplement: Supplementary file 5 — Authors’ original file for figure 4 [file 12284_2012_12_MOESM5_ESM.tiff]

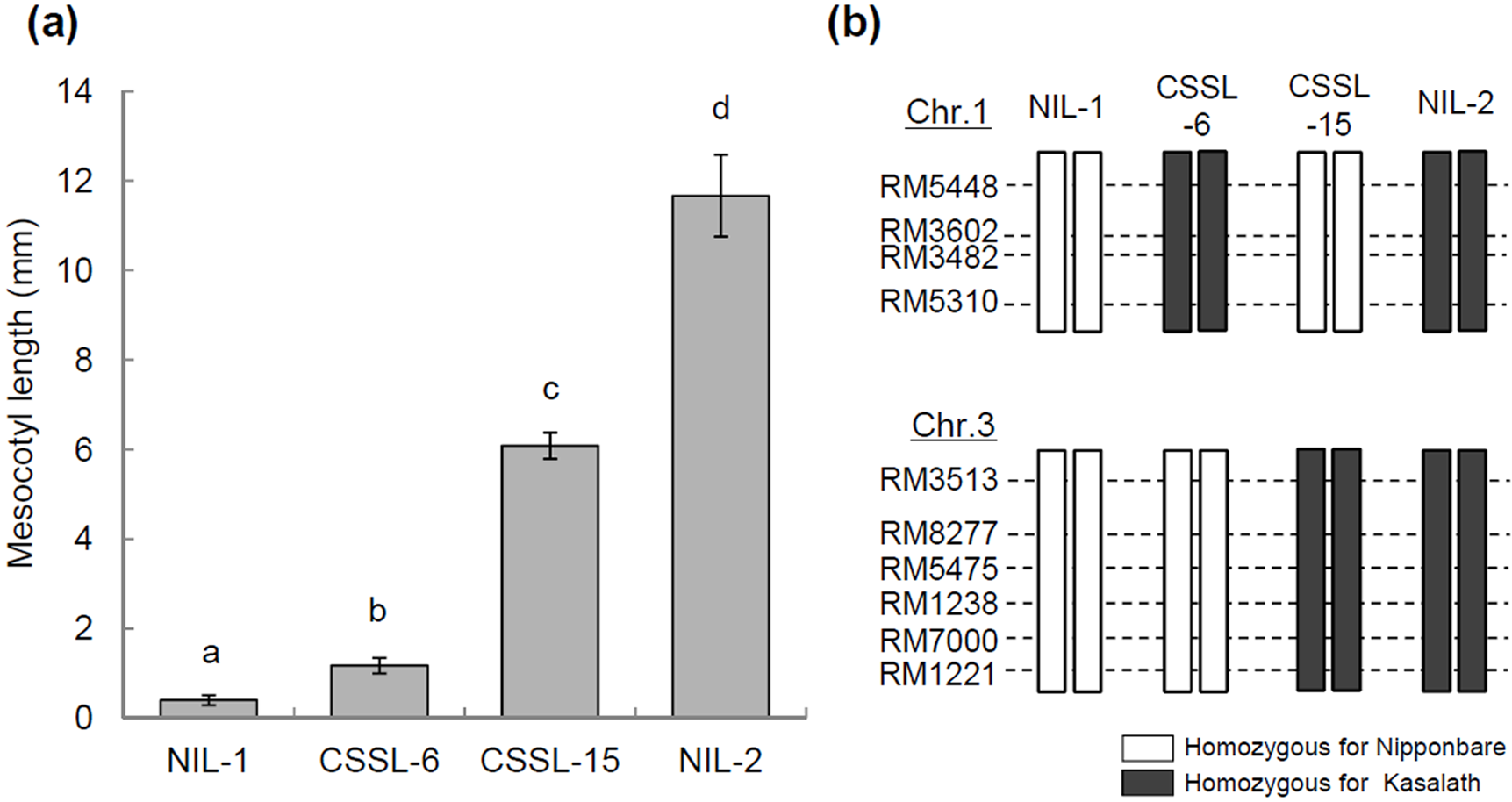

Supplement: Supplementary file 6 — Authors’ original file for figure 5 [file 12284_2012_12_MOESM6_ESM.tiff]

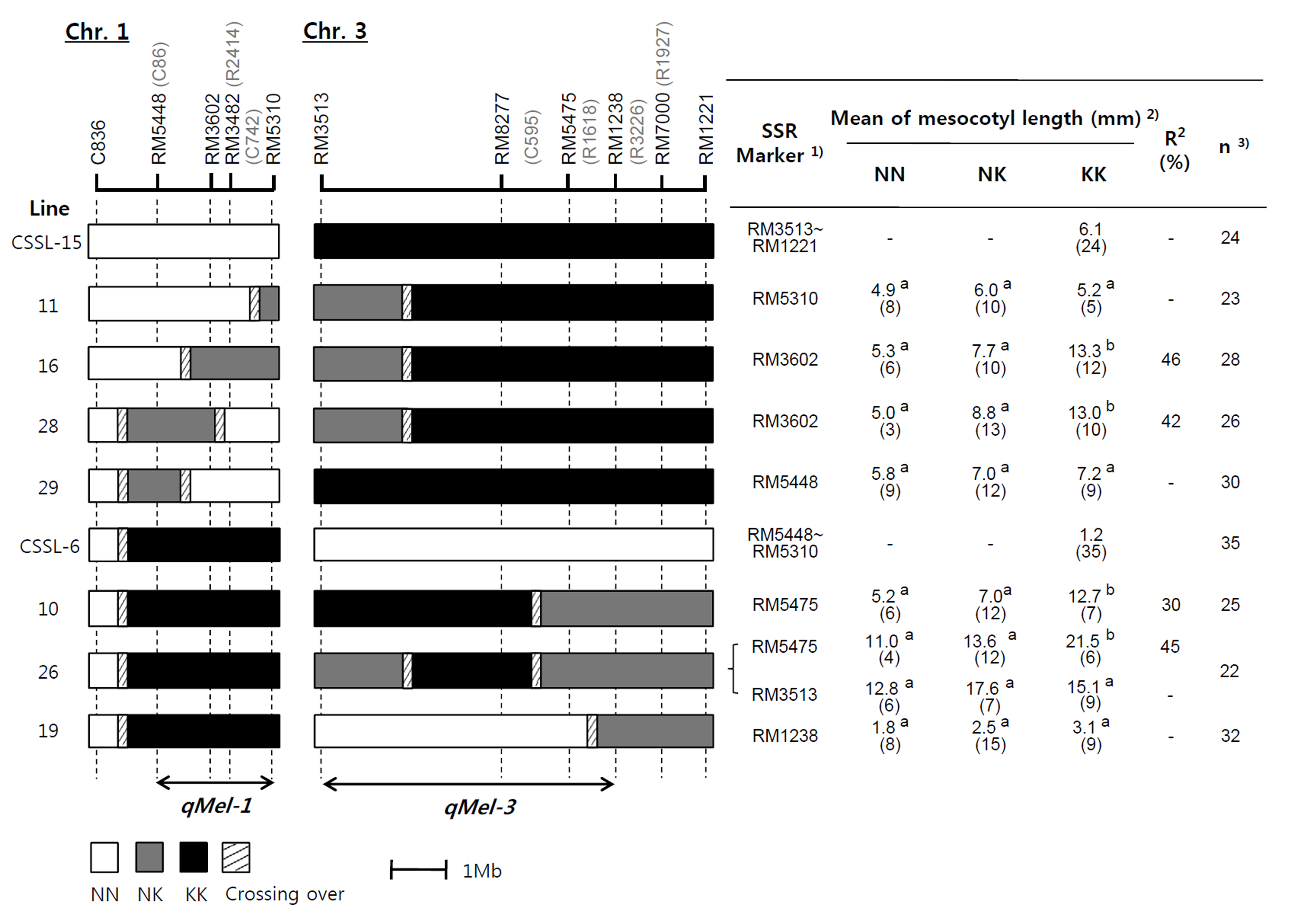

Supplement: Supplementary file 7 — Authors’ original file for figure 6 [file 12284_2012_12_MOESM7_ESM.tiff]
